# Prevalence of current patterns and predictive trends of multidrug-resistant *Salmonella* Typhi in Sudan

**DOI:** 10.1186/s12941-017-0247-4

**Published:** 2017-11-14

**Authors:** Ayman A. Elshayeb, Abdelazim A. Ahmed, Marmar A. El Siddig, Adil A. El Hussien

**Affiliations:** 1 Faculty of Science, University of Khartoum, Khartoum, Sudan; 2 Albaha University, Al Bahah, Saudi Arabia

**Keywords:** Antibiotics, Ciprofloxacin, *Salmonella* Typhi, Prediction, Resistance

## Abstract

**Background:**

Enteric fever has persistence of great impact in Sudanese public health especially during rainy season when the causative agent *Salmonella enterica serovar* Typhi possesses pan endemic patterns in most regions of Sudan - Khartoum.

**Objectives:**

The present study aims to assess the recent state of antibiotics susceptibility of *Salmonella* Typhi with special concern to multidrug resistance strains and predict the emergence of new resistant patterns and outbreaks.

**Methods:**

*Salmonella* Typhi strains were isolated and identified according to the guidelines of the International Standardization Organization and the World Health Organization. The antibiotics susceptibilities were tested using the recommendations of the Clinical Laboratories Standards Institute. Predictions of emerging resistant bacteria patterns and outbreaks in Sudan were done using logistic regression, forecasting linear equations and in silico simulations models.

**Results:**

A total of 124 antibiotics resistant *Salmonella* Typhi strains categorized in 12 average groups were isolated, different patterns of resistance statistically calculated by (y = ax − b). Minimum bactericidal concentration’s predication of resistance was given the exponential trend (y = n e^x^) and the predictive coefficient R^2^ > 0 < 1 are approximately alike. It was assumed that resistant bacteria occurred with a constant rate of antibiotic doses during the whole experimental period. Thus, the number of sensitive bacteria decreases at the same rate as resistant occur following term to the modified predictive model which solved computationally.

**Conclusion:**

This study assesses the prediction of multi-drug resistance among *S.* Typhi isolates by applying low cost materials and simple statistical methods suitable for the most frequently used antibiotics as typhoid empirical therapy. Therefore, bacterial surveillance systems should be implemented to present data on the aetiology and current antimicrobial drug resistance patterns of community-acquired agents causing outbreaks.

**Electronic supplementary material:**

The online version of this article (10.1186/s12941-017-0247-4) contains supplementary material, which is available to authorized users.

## Background

Globally the burden of typhoid fever victims are 20 million as a main issue of morbidity and mortality in developing countries [1, 2]. In Sudan, approximate numbers of typhoid infection is increasing during rainy season in association with malaria incidents [3–5]. *Salmonella* Typhi as causative organism has rapidly gained resistance to antibiotics like ampicillin, chloramphenicol and cotrimoxazole, and also to previously effective drugs namely fluoroquinolone [6]. Resistance to the antimicrobial agents such as trimethoprim–sulfamethoxazole, chloramphenicol and amoxicillin has been increasingly reported among *S.* Typhi isolates. In addition, quinolone resistance has been reported from Southeast Asia and the Indian subcontinent. Determining the patterns of antimicrobial resistance is essential in recommending effective treatment for bacterial infection [7, 8]. The incidence of multidrug resistant *S*. Typhi was found to be increasing globally and has been a scourge for those afflicted with enteric fever all over the world, while there are many reports noting decline in susceptibility to ciprofloxacin [9]. Resurgence of resistant Salmonella well-known as increasing to the amount of multi drug resistant strains against nalidixic acid even though the isolates were sensitive to ceftriaxone and ciprofloxacin [10]. Until now, Ciprofloxacin is a promising antibiotic against numerous bacterial infections, for its ability to penetrate into the macrophages and to kill multidrug-resistant strains [11]. On the other hand, emergence of resistant Salmonella strains against fluoroquinolone has been reported [12–14]. Since fluoroquinolone has broad spectrum, potency, and oral efficacy, it could be useful for oral treatment of general bacterial infections [15]. Therefore, it is necessary for susceptibility pattern of the particular strain isolated from patient to be determined by the sensitivity test in vitro [16]. Variation in patterns of susceptibility of *S*. Typhi is important to monitor it and provide suitable guidelines on fluoroquinolone treatment given as oral tablets that have better absorption from the gastrointestinal tract after oral administration [17]. Routine investigation for the antibiotics members of fluoroquinolone MICs in patients presenting with invasive Salmonella infections should be done [18]. However, revealing increased resistance to ciprofloxacin (MICs, 0.125–1 µg/mL/L) had emerged and become endemic in South and South-East Asia, also such strains have also been described from other parts of the globe [19, 20]. Accordingly, there is treatment collapse of fluoroquinolones in patients infected with these organisms, because isolates with reduced susceptibility to fluoroquinolones might become highly resistant to sequential increasing of mutations in topoisomerase genes [21, 22]. The prediction of drug efficiency started by the use of uncomplicated screening tools such as implying antibiotic discs are of great value [23]. For predicting resistance and outbreaks problems, it is essential to create the mathematical time-series incorporate any sort of statistical trend, otherwise by definition forecasting would be impossible [24]. The application of in silico models’ parameters that assessed the estimated efficiency of any antibiotic therapy in specific bacterial population dynamics, required the computational simulation and mathematical data analysis. It also required the possibility to monitor the outbreaks occurrence on the suspected environment and to mark the actual MIC spread values [25].

### Objectives

This work was conducted to evaluate the recent efficiency of common antibiotics used against *Salmonella* Typhi in Sudan and predict the future trends and fluctuations of susceptibility using inexpensive materials and simple statistical methods to support the decision for antimicrobial choice and dosage especially during rainy season and outbreaks.

## Methods

### Isolation of *Salmonella* Typhi

Environmental samples collected from wastewaters of Omdurman Military Hospital and Soba Stabilization Station ponds, showed positive numbers of Multidrug *Salmonella* Typhi (n = 128). Laboratory investigations were done immediately after collections include; cultural characteristics and biochemical tests were performed according to the recommendations of [26] standards. Serotyping was done using polyvalent O-anti sera A–G and H (flagellar)-anti sera formula.

### Antibiotic susceptibility

The antimicrobial susceptibilities of the isolated strains were tested following the Clinical Laboratory Standards Institute guidelines [27]. The experimented antibiotics were those commonly used for Gram negative bacteria by Kirby Bauer disk diffusion susceptibility test which include; tetracycline (TE_30mcg_), ofloucin (OF_5mcg_), cefroxin (CXM_30mcg_), cotrimoxazole (COT_25mcg_), amoxyclar (AMC_30mcg_), gentamycin (GEN_30mcg_) and ciprofloxacin (Cip_5mcg_). The discs were placed on the top surface of the agar plates, inoculated with *Salmonella* Typhi and incubated overnight at 37 °C. The diameters of inhibition zones were calculated using reference tables to determine whether the bacteria are sensitive (S), intermediate (I) or resistant (R) to the certain antibiotic. A standard *E. coli* strain ATCC 25922 was also tested for quality control [28].

### Determination of minimum inhibition concentration (MIC)

Bacterial suspensions were made in sterile nutrient broth by colonies from a pure cultures and the turbidity adjusted to 0.5 McFarland standards to make a concentration of about 10^7^ CFU/mL. Sensitivity to ciprofloxacin was evaluated by the macro-dilution test according the criteria stipulated by the [29] with serial Ciprofloxacin dilutions of 8, 16, 32, 64 and 128 µg/mL respectively. Results were recorded as minimum inhibitory concentrations (MIC). A standard *E. coli* strain ATCC 25922 was also tested for quality control.

### Determination of minimum bactericidal concentration (MBC)

Following the MICs determination, 100 μL were aspirated from the wells of the micro-plates that showed growth inhibition and then inoculated on the top surface of plates containing Mueller–Hinton agar medium. Plates were incubated overnight and examined visually for bacterial growth. A standard strain *E. coli* ATCC 25922 was also tested for quality control.

### Probability of antibiotics resistance (%)

To determine the thresholds of bacteria resistance to ciprofloxacin, statistical probabilities for MBCs breakpoints were done by trending normal probability plot.

### Prediction of antibiotics resistance

Time-series prediction (calculated from the current susceptibility pattern) was employed by using simple statistical systems (regression and simple moving average) for the temporal variations in *Salmonella* Typhi sensitivity or resistance towards antibiotics and resulted in (Tables 2 and 3). In silico model of outbreak was created for solving the prediction of bacteria resistance due to periodic samples collections [30].

## Results

### Isolation and identification of *S. typhi*

From the recovered bacteria species, 128 were identified as *Salmonella* Typhi, the isolates stereotyping showed that; 9 groups (75%) had the antigenic formula O:9(D_1_) for *Salmonella* Typhi, 3 groups (25%) had the formula *Salmonella enteric* Paratyphi A 1,2,12 a [1, 5] and *Salmonella enterica* Paratyphi C1 6,7,[Vi] c 1,5 respectively.

### Antibiotics susceptibility test

Different patterns of susceptibility were shown by the 12 *S. typhi* isolated groups for their response against the seven tested antibiotics. *Salmonella* Typhi defined as multi drug resistant when isolate resist two or more tested antibiotics, exceptions were COT_25mcg_, GEN_30mcg_ and Cip_5mcg_ which were susceptible. However, three isolates (S3, S4 and S6) had intermediate resistance to GEN_30mcg_. Eight isolates were resistant to CXM_30mcg_.

Antibiogram or (in vitro-sensitivity) showed susceptibility patterns of *Salmonella* Typhi isolated groups that have developed resistance against most antibiotics (multi drug resistant) including cotrimoxazole, gentamycin and ciprofloxacin and interpreted as MIC ≥ 32 µg/mL.

### Minimum inhibitory concentration (MIC)

The MIC of ciprofloxacin against the *S.* Typhi isolated strain and the control *E. coli* strain indicated that two *S.* Typhi *Dr11* and S8 were capable to tolerate ciprofloxacin as high as 32 µg/mL, five (S3, S6, S7, S9 and S10) can grow in 16 µg/mL and four isolates (Dr14, S1, S4 and S5) showed no growth at concentrations higher than 8 µg/mL while isolate S2 could not tolerate any of the tested ciprofloxacin concentrations. Therefore, any isolated strain that showed resistance to ciprofloxacin higher than 16 µg/mL was considered as resistant isolate. The means of (33.0%) of the isolates found to be multi-drug resistant strains and categorized in 8 main groups namely (S. Typhi 16, S. Typhi 32, S.7 16, S.7 32, Dr11 16, Dr11 32, Sal C 16, Sal C 32). The Minimum Inhibitory Concentrations MICs were determined at 16 and 32 µg/mL concentrations, and (67.0%) isolates that resist ciprofloxacin as minimum bactericidal concentration (MBCs).

### Minimum bactericidal concentration (MBC)

The MBC breakpoints of the 12 *S.* Typhi and the control *E. coli* can tolerate 32 µg/mL antibiotics indicating an emergence of resistance against this antibiotic that should have more attention.

### Probabilities of antibiotics resistance occurrence among (MBCs)

Statistical analysis between antibiotics’ concentrations µg/mL and MBCs breakpoints was done by MBCs trending plot for the probability of resistance occurrence.

According to the data obtained from the tested patterns of susceptibility, the probability of an isolate to develop resistance increases with antibiotics concentration increasement as shown in (Table 1). The determination of Spearman coefficient (R-square) was calculated R^2^ = 0.8568 and its corresponding p value was founded (p = 0.022038522). For the estimation of the regression lines, each of the data points was plotted and weighted by the inverse of the variance and the rate of regression (y = 3.0919e^0.0162x^).

**Table 1:** Probabilities of multi drug resistance occurrence

Interpretation	Ciprofloxacin concentration µg/mL	Probability of resistance occurrence %
Control	0.0	0
S≥	8.0	25
I=	16.0	42
R≤	32.0	58
R≤	64.0	75
R≤	128.0	92

The plotted graph showed the percentage probability of MBCs to resist antibiotics concentrations and their empirical observations were solved by

1 $$\left( {{\text{y }} = {\text{ nx}}{-}{\text{b}}} \right).$$

The Spearman coefficient of determination was calculated R^2^ = 0.8381 and it corresponds significantly to the rate of resistance occurrence by (y = 0.0235x − 0.0411).

### Prediction of antibiotics susceptibility trends

The prediction of possible resistance occurrence was statistically estimated by regression and simple moving average for certain period of incubation with antibiotics. The increasing numbers of MBCs equal to the probability of more cells grown with antibiotics and develop resistance during the first 12 h and calculated for the next 7 days of incubation period as shown in (Tables 2 and 3).

**Table 2:** Prediction of antibiotics resistance interpreted of 16 µg/mL

Incubation	*E. coli*	Dr 11	Dr 14	S1	S2	S3	S4	S5	S6	S7	S8	S9	S10
Actual 12 h	6	9	6	5	4	3	3	5	6	6	7	9	8
Day 1	7	9	5	6	4	3	5	6	6	6	7	9	9
Day 2	8	8	7	5	5	4	6	6	7	7	8	10	11
Day 3	8	9	7	6	5	4	7	7	7	7	9	12	12
Day 4	9	10	8	7	5	5	6	7	7	7	9	12	12
Day 5	9	11	8	7	6	5	6	8	8	8	11	13	13
Day 6	9	12	8	8	7	6	8	9	8	8	13	14	13
Day 7	9	13	8	8	7	6	8	9	9	8	16	15	14

Predicting low-level resistance of antibiotics at 16 µg/mL as intermediate dose among *S.* Typhi is an indicator of treatment failure in the nearest future

**Table 3:** Prediction of antibiotics resistance interpreted of 32 µg/mL

Incubation	*E. coli*	Dr 11	Dr 14	S1	S2	S3	S4	S5	S6	S7	S8	S9	S10
Actual 12 h	1	8	3	1	2	1	1	2	2	2	5	6	4
Day 1	1	9	6	2	2	2	1	2	3	2	5	6	5
Day 2	1	10	7	4	2	2	1	2	3	3	6	7	5
Day 3	2	10	8	6	3	2	1	3	3	3	6	7	6
Day 4	2	10	9	7	4	2	2	3	3	3	7	8	7
Day 5	3	10	10	8	6	3	2	3	4	3	7	8	7
Day 6	4	11	10	9	7	4	3	3	4	3	8	9	7
Day 7	5	12	11	10	8	6	3	4	5	4	8	10	8

Predicting high-level resistance of antibiotics at 32 µg/mL as resisted dose among *S.* Typhi is an indicator of total treatment failure. It is clear that both Tables 2 and 3 showed the numbers of resisted colonies have daily developed increasing resistance with no specific pattern.

Minimum bactericidal concentration’s predication of *E. coli* ATCC 25922 resistance was given by (y = 6.2391e^0.0513x^) and the coefficient (R^2^ = 0.7). *Salmonella* Typhi, (Dr11, Dr14, S1…S10) were obtained by the exponential trend

2 $$\left( {{\text{y }} = {\text{ n e}}^{\text{x}} } \right)$$

and the coefficient R^2^ > 0 < 1 are approximately alike.

The bacteria resistance after long term of incubation period with antibiotics was predicted by (y = n e^x^), where (n) is the numbers of colonies (MBCs) and (e^x^) is the exponent positive integers which corresponds to increasing probabilities of resistance incident at certain time. The predictive coefficient R^2^ value for each isolate of antibiotics concentrations (16 and 32 µg/mL) are shown in (Figs. 3 and 4).

The solution of the linear equation (y = n e^x^) on plotted Excel sheet solved the prediction of long term incubated bacteria with antibiotics. The predictive patterns of all isolated strain showed increasing in their exponential trends of antibiotics concentrations of 16 and 32 µg/mL which correlate to the increasing probabilities of resistance occurrence (Table 4), the table also showed high values of coefficient R^2^ > 0.5 and this indicates how well data fit the statistical model.

**Table 4:** Results of linear predictive patterns of resistance in *Salmonella* Typhi

Sample	16 µg/mL		32 µg/mL	
	Exponential trend	Coefficient	Exponential trend	Coefficient
*E. coli* ATCC 25922	y = 6.2391e^0.0513x^	R^2^ = 0.7208	y = 0.5978e^0.2636x^	R^2^ = 0.9455
Dr11	y = 7.621e^0.0604x^	R^2^ = 0.7886	y = 8.0752e^0.0458x^	R^2^ = 0.9347
Dr14	y = 5.2505e^0.0676x^	R^2^ = 0.7903	y = 3.7532e^0.1528x^	R^2^ = 0.7738
S1	y = 4.5725e^0.0717x^	R^2^ = 0.9591	y = 1.153e^0.3069x^	R^2^ = 0.8695
S2	y = 3.2991e^0.0977x^	R^2^ = 0.9339	y = 1.1018e^0.2501x^	R^2^ = 0.9066
S3	y = 2.7596e^0.1016x^	R^2^ = 0.9254	y = 0.7688e^0.2367x^	R^2^ = 0.9544
S4	y = 3.6258e^0.1079x^	R^2^ = 0.6979	y = 0.7328e^0.172x^	R^2^ = 0.9362
S5	y = 4.8046e^0.0834x^	R^2^ = 0.958	y = 1.5462e^0.1135x^	R^2^ = 0.7652
S6	y = 5.5536e^0.0552x^	R^2^ = 0.8698	y = 2.0479e^0.1038x^	R^2^ = 0.8484
S7	y = 5.7606e^0.0459x^	R^2^ = 0.884	y = 1.8061e^0.0911x^	R^2^ = 0.9553
S8	y = 5.7004e^0.1168x^	R^2^ = 0.9397	y = 4.5789e^0.0745x^	R^2^ = 0.9477
S9	y = 8.0663e^0.0787x^	R^2^ = 0.9797	y = 5.2388e^0.0759x^	R^2^ = 0.886
S10	y = 7.7298e^0.0796x^	R^2^ = 0.9237	y = 3.9538e^0.0955x^	R^2^ = 0.9566

### In silico simulation for predicting patterns of outbreak

For long term predicting of MDR *Salmonella* Typhi in certain period (5 years) for outbreak purposes, a monitoring simulated system was created from bacteria collected during study period to predict population dynamics especially during rainy season and/or outbreak (Additional file 1). In ideal microbial community dynamics, the normal bacteria growth rate is given by

3 $${\text{N}}_{\text{t}} = {\text{ N}}_{0} * \, \left( { 1 { } + {\text{ r}}} \right)^{\text{t}} ,$$

where N_t_: bacterial amount at time t, N_0_: bacterial amount at time 0, r: growth rate and t: time passed.

Introducing antibiotic to the population gives different interactions of *Salmonella* Typhi and become

4 $$\left[ {\left( {\text{N}} \right) = {\text{Replicated population}} + {\text{Resistance}} - {\text{Mortality}}} \right].$$

Since multidrug resistant *Salmonella* Typhi (N) affected by antibiotics (∆) and denoted by (α) = [range of C_min_ up to C_max_/MIC], in time (t), where: N = variations in concentrations of Salmonella population dynamic = (N_0_ − N_t_), t = variations time (t_0_ − t_1_). The MIC and the (α) target values for these indices are 16 and 32, respectively and become (C_16_, C_32_). The (α) elimination of the initial concentration of antibiotics affected bacteria [resistant − mortality (s − m)] and E = prediction of multidrug Salmonella resistance outbreak where (N, t ≠ 0).

Changes in these populations depend only on the net growth rate at time (t). Where a, b are positive constants and the initial bacteria N at time (0) = N_0_, If a > b the bacteria grow exponentially, if a < b the bacteria die out as shown in (Table 5)

**Table 5:** Outbreaks’ simulation system parameters

Description	Parameter	Estimated value	Simulated value	Units
Initial population	N_0_	y = n e^x^	1.0000	Cell/mL
Bacteria replication time	t	(t > 0)	30	min
Bacterial replication rate	r	ln x^(+a)^	100	
Mortality of bacteria	m	ln x^(−b)^	25	
Probability to resist antibiotics	∆		2.00	
Antibiotics influence dose	α	C_16_, C_32_	2.0 and 5.0	µg/mL
Final population	N_t_	ln x^(a−b)^	2.99573	Cell/mL
Resisted individuals (survived)	N	ln (x)	12	
Prediction of outbreaks	E		− 13.00%	

It was assumed that resistant bacteria occurred with a constant rate during the whole experimental period. Since different sub-populations resemble patterns of infection-causing inoculums (one highly susceptible with MIC = 2.0 µg/mL, and one more resistant with an MIC = 5.0 µg/mL) that differ not only in EC_max50_ but also in the slope of their relationship to drug concentrations which is simulated as the outcome of treatment regimen illustrated in (Fig. 5).

## Discussion

**Fig. 1:**
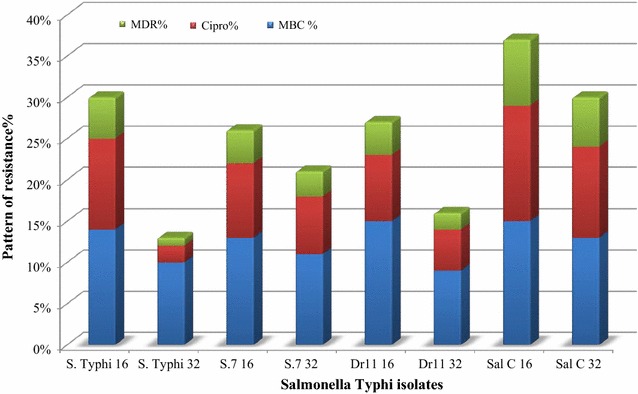
Antibiogram criteria for multi-drug resistant strains

**Fig. 2:**
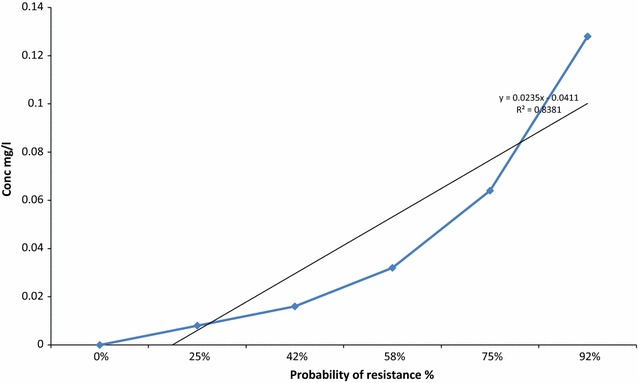
Antibiotics normal probability plot

**Fig. 3:**
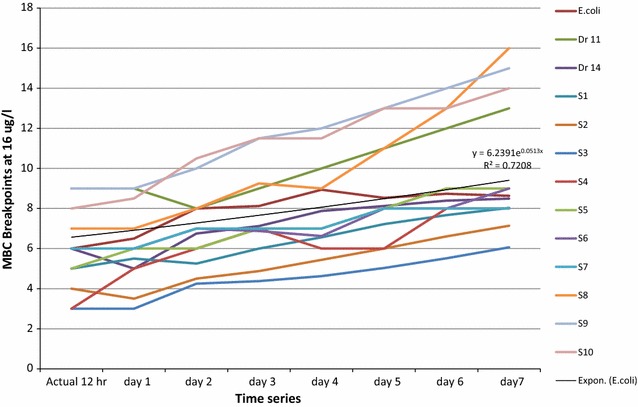
Prediction of antibiotics MBC resistance’s trends at 16 µg/mL

**Fig. 4:**
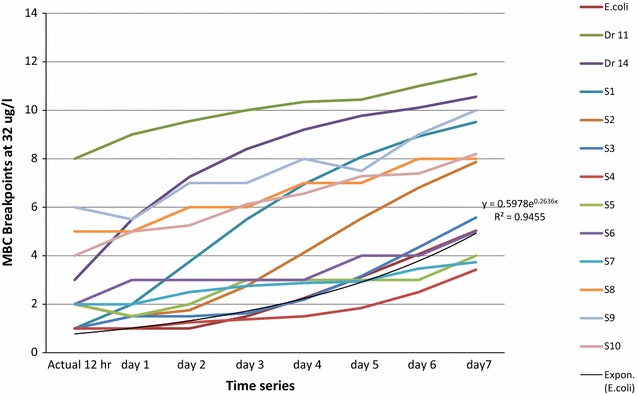
Linear predicition of antibiotics MBC resistance’s trends at 32 µg/mL

**Fig. 5:**
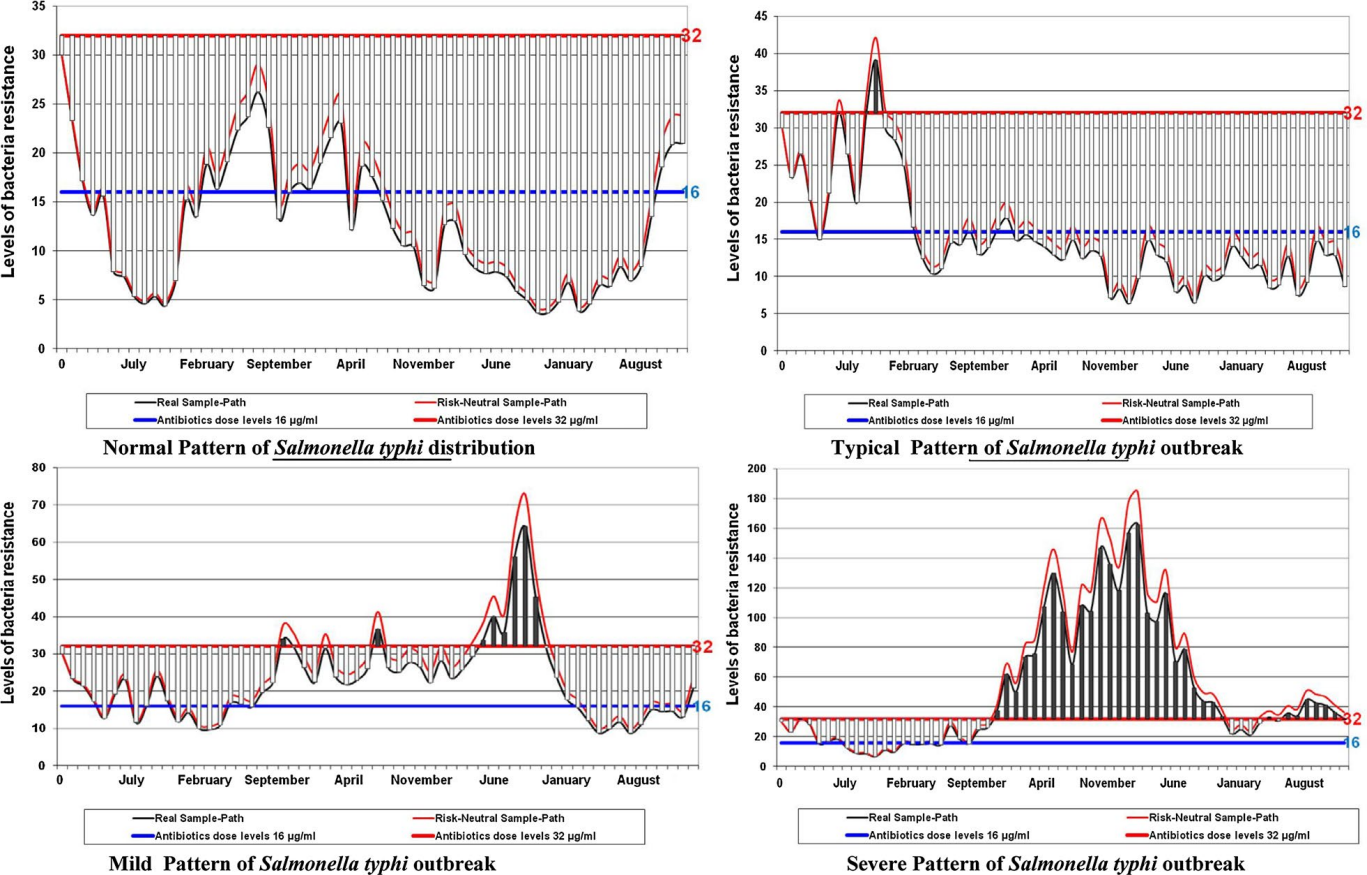
In silico simulations of MDR *Salmonella* Typhi outbreaks. The in silico monitoring system is a computational program based on Microsoft Excel sheet describes the predicting statistics was found to be suitable for monitoring the seasonal typhoid incidents during the outbreaks

The increasing of multi drugs resistant bacteria in Sudan is a real issue and a major concern for all health authorities [31]. Although massive reports mentioned the increasing of resistance globally but the current patterns of MDR *Salmonella* spp. in Sudan is acceptable [32]. For effective measuring patterns and predicting of bacteria resistance, a microbial monitoring system was launched for Soba and Omdurman Military Hospital Stabilisation Stations were *Salmonella* Typhi originated from infected humans and survive during treatment at stations’ ponds to invade the environment [33–35] have previously reported this epidemiological issue. The system was effectual for collections of samples especially during rainy seasons when outbreaks were expected; these agreed with [36] who also highlighted the hazard of scattering resistant Salmonella strains from wastewater to the environment and infect humans again. Fourth generation Fluoroquinolone antibiotics are commonly used in Sudan to treat a variety of illnesses such as respiratory and urinary tract infections [37–39]. These antibiotics were tested and the proposed breakpoints were calculated with a standard *E. coli* ATCC 25922 reference as the Clinical and Laboratory Standards Institute (CLSI) recommendation for quality control, this in agreement with [28, 40, 41]. Interpretation of antibiotics’ susceptibility for *Salmonella* Typhi include ciprofloxacin, gemifloxacin, levofloxacin, moxifloxacin, norfloxacin, and ofloxacin showed sensitive pattern of cotrimoxazole, gentamycin and ciprofloxacin to all isolated strain (Fig. 1) and resistance to some of the others, this agreed [42–44] who reported similar results of Ciprofloxacin susceptibility pattern for *Salmonella* Paratyphi indicating their sensitivity as antimicrobial. Statistical explanation of antibiogram indicated regular pattern of susceptibility by determining MICs and MBCs breakpoints of the isolated strains at (16 µg/mL) as threshold, the result agreed [45] and explained by [46, 47], when the isolated strain become more tolerant to the antibiotics they showed higher MICs without new mutation after 8 µg/mL. Moreover, probability trends in the MIC or its resultant MBC distribution above the threshold may not be observed whereas the distribution to the left of the threshold is not expected to change over time [48]. When such changes in the resistant isolated strain are expected then the ordinal or quantitative scale of distribution trends needs to be considered as confirmed by [49]. The probability of *Salmonella* Typhi resistance may occur during the course, and it can be plotted as linear equation (Eq. 1), where the coefficient of isolated strain found to be resistant as calculated in (Fig. 2), [50] reported the application of related antibiotics such as (beta-lactam antibiotics and macrolides) and the log odds of resistance could be modelled in the course of linear regression where a strong linear and statistically significant relationship is confirmed. The in vitro prediction of resistance trends demonstrated that the scale of the impact was relatively more influential for cotrimoxazole, gentamycin and ciprofloxacin, (Tables 2 and 3), [51] identifying the MIC of antimicrobial agent is an effective assessment for clinical success using breakpoints determination for predicting and detecting the probability of resistant populations during specific timeline. The emergence of resistance pattern could be predicted by changing the values from actual to forecast in the time-line of the experiment (Figs. 3 and 4) depending on bacteria exponential growth during antibiotics course (Eq. 2), reports from [52–54] confirmed that; various antimicrobial agents are related to the emergence of resistance during prolonged therapy. Consequently, isolated strains that are originally susceptible may become resistant within three to 4 days after the beginning of treatment [55]. Conversely, the previous records of rapid emergence of resistance to recently introduced antibiotics indicates that even new families of antibiotics will have a short life expectancy [56], to avoid this critical issue, a predictive system was created and applied during collection of samples. Earlier information of antibiotic’s efficiency in treating a bacterial infection can potentially play a major role in controlling serious outbreaks [57, 58], the long-term prediction of bacterial population and antibiotics dynamics was modelled (Eqs. 3 and 4) using such information. Therefore, the purpose of in silico simulation models as shown in (Table 4) for this research is to observe the seasonal typhoid incidents computationally because it is essential to predict the outcome of their trended outbreaks as reported by [59]. To control a successful strategy of a typical disease scenario (Fig. 5), the measure plan is to eliminate the causative bacteria before it has the opportunity to spread and infect more population. However, modelling is essential in drug development for certain pathogenic population, and it contains many complex processes that require robust of basic procedures for predicting clean data, fitting computing platforms, sufficient resources, and valuable communication. Absolutely, the application of model-based approaches for drug development and for maximizing the clinical potential of drugs is a complex and developing field, precisely in silico models that have considerable attention during outbreaks because they are essential for connecting pharmacokinetics information and clinical outcomes [60].

## Conclusion

The present patterns of ciprofloxacin susceptibility are in normal ranges. This study assesses the prediction of multi-drug resistance among *S*. Typhi isolates by applying low cost materials and simple statistical methods suitable for the most frequently used antibiotics as experimental therapy. Consequently, bacterial surveillance systems were implemented to present data on the aetiology and current antimicrobial drug resistance patterns of community-acquired agents causing bacteraemia and outbreaks.

## Additional file

**Additional file 1.** Prediction of MDR *Salmonella* Typhi Simulations.
